# Reconstruction of a metacarpal head defect due to bite injury: two case reports

**DOI:** 10.1080/23320885.2018.1509717

**Published:** 2018-10-25

**Authors:** Akito Nakanishi, Kenji Kawamura, Shohei Omokawa, Takamasa Shimizu, Yasuhito Tanaka

**Affiliations:** a Department of Orthopaedic Surgery, Nara Medical University, Nara, Japan;; b Department of Hand Surgery, Nara Medical University, Nara, Japan

**Keywords:** Vascularised osteoarticular flap, metacarpophalangeal joint, osteomyelitis, bite injury

## Abstract

We present two rare cases of acute osteomyelitis after bite injury that were reconstructed with a third metacarpal base osteoarticular flap and a vascularised medial femoral trochlea osteocartilaginous flap. The outcomes show that a vascularised osteoarticular flap is a good treatment option for a metacarpal head defect.

## Introduction

The metacarpophalangeal (MCP) joint is important for finger function because their average active range of motion accounts for 36% of total finger motion [1,2]. However, there are few salvage procedures available for a posttraumatic intra-articular injury of the MCP joint, except joint arthrodesis and prosthetic arthroplasty for MCP joint reconstruction. To reduce the development of secondary degenerative arthritis, restoration of the articular surface of the MCP joint is preferable [2]. Here, we report two rare cases of acute osteomyelitis after a cat bite injury and a fight bite injury that were reconstructed with a third metacarpal base osteoarticular flap and a vascularised medial femoral trochlea osteocartilaginous flap.

## Case reports

### Case 1

**Figure 1.**
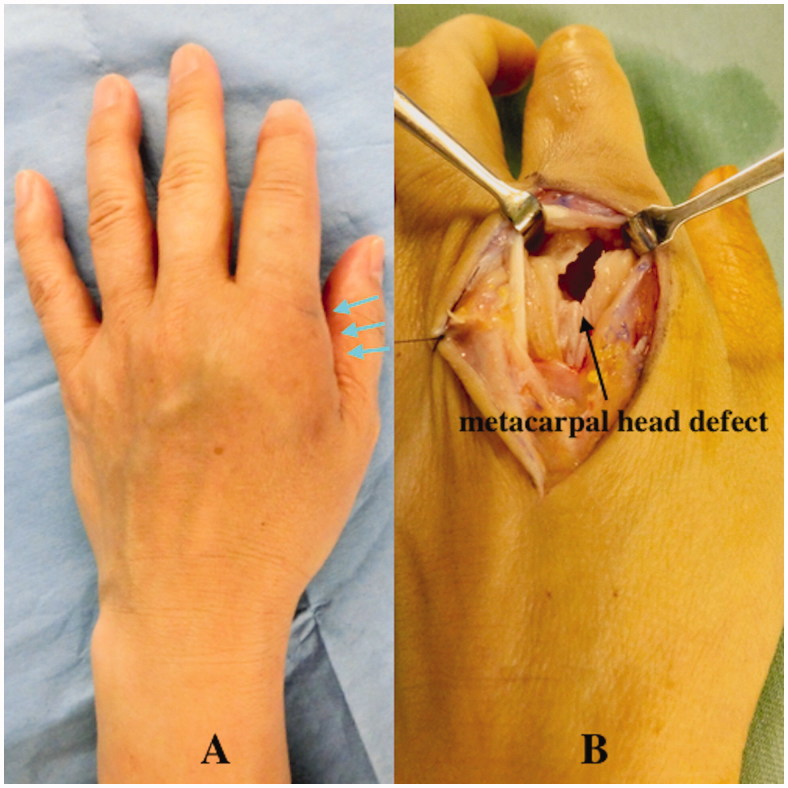
Case 1. (A) Local findings at admission. Arrows show noticeable swelling and erythema on the dorsum of the second finger metacarpophalangeal joint. (B) Partial defect of the second metacarpal head due to infection.

A 41-year-old left hand-dominant female visited the emergency room of another hospital with a small (5-mm long) laceration wound on the dorsum of the left second MCP joint due to a bite injury from her pet cat. A physical examination showed a simple laceration wound with mild tenderness, and an initial X-ray examination was not performed. A simple wound dressing was performed and the patient was discharged with a prescription for regular oral antibiotics. Three weeks later, the patient visited our hospital with noticeable swelling, erythema, and tenderness on the dorsum of the MCP joint of her second finger (Figure 1(A)). Body temperature was 37.6 °C. Laboratory tests revealed elevated white blood cells (11.25 × 10^9^/L) and C-reactive protein (12.05 mg/L). X-ray showed focal osteopenia at the second metacarpal head. We diagnosed acute osteomyelitis in the second metacarpal bone and quickly performed surgical debridement. During surgery, a partial lesion of the second metacarpal head due to infection was found, and necrotic and infectious bone was removed completely (Figure 1(B)). An intraoperative Gram-stain wound culture did not reveal any bacteria due to the effect of the oral antibiotics from the first hospital.

**Figure 2.**
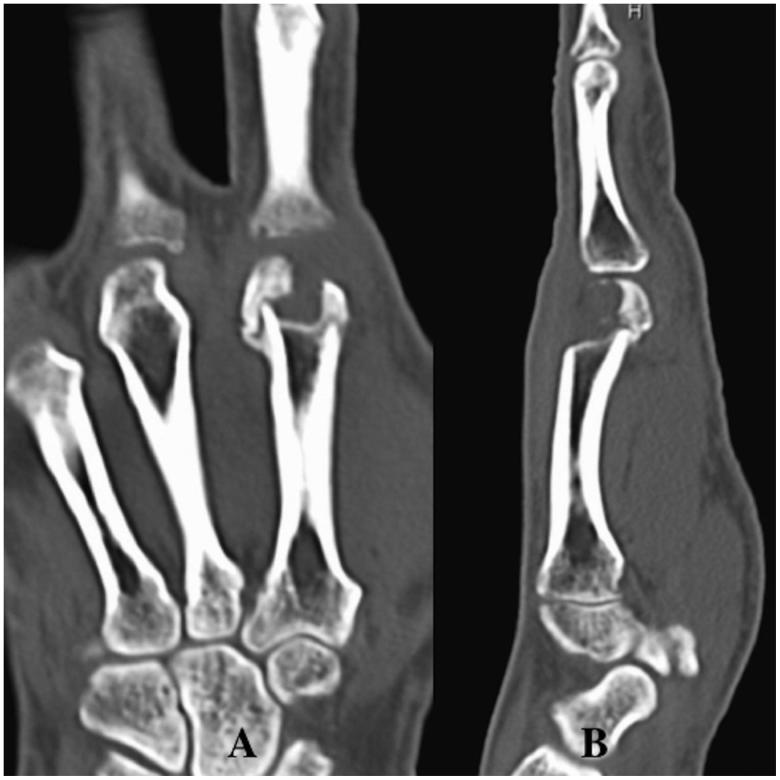
Case 1. (A and B) CT images showing the second metacarpal head defect.

**Figure 3.**
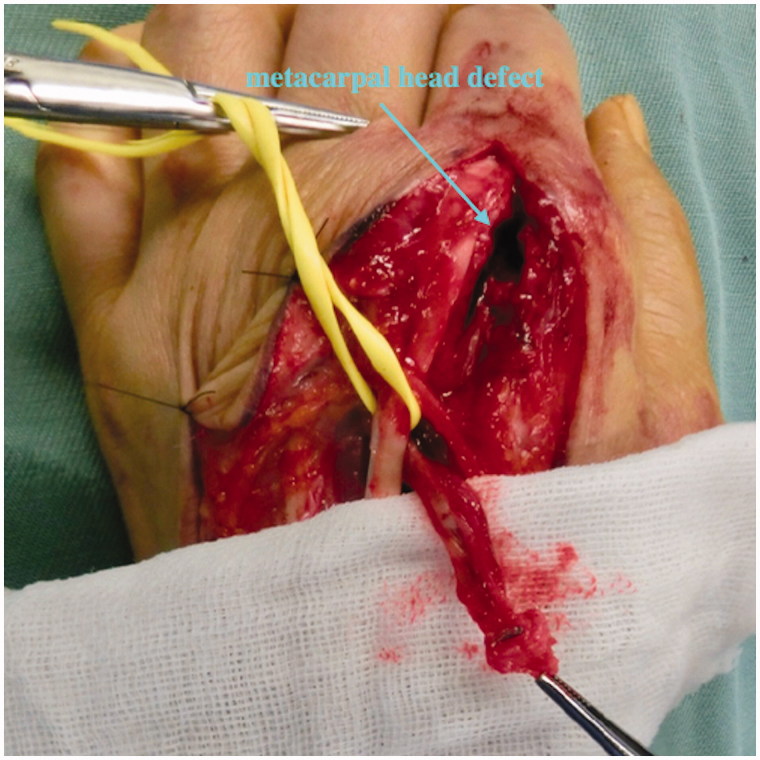
Case 1. A third metacarpal base osteoarticular flap was harvested.

**Figure 4.**
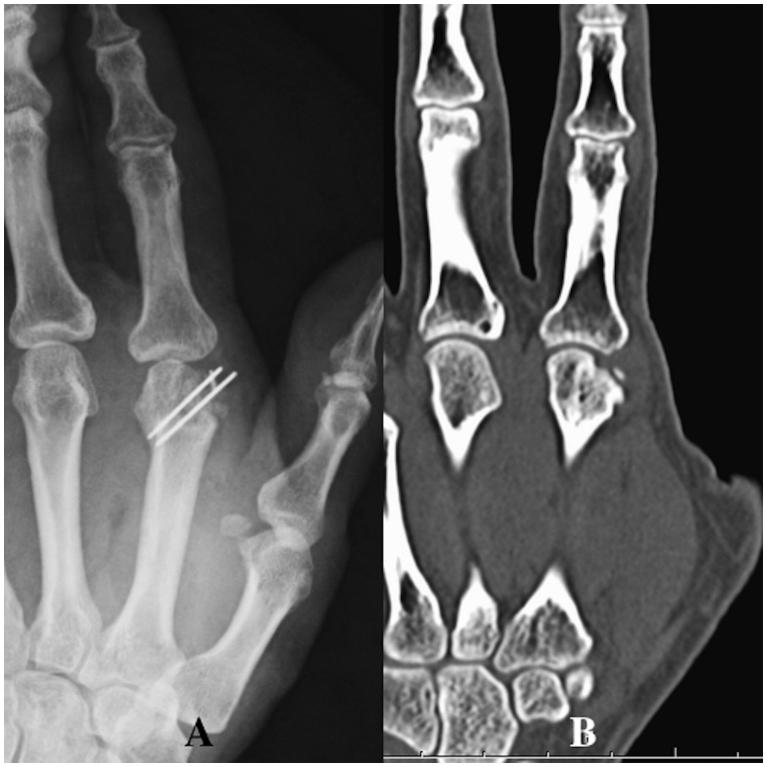
Case 1. (A) The vascularised bone fragment was fixed with K-wires. (B) Bone union of the vascularised bone graft was obtained at the articular surface of the MCP joint at final follow up.

The patient was treated with intravenous systematic antibiotic therapy, including a second-generation cephalosporin. One month after the initial surgery, her inflammatory symptoms improved and she showed complete resolution of the osteomyelitis. However, she could not move her right second finger MCP joint without pain due to the second metacarpal head defect (articular defect size: 13 × 10 mm) (Figure 2). The motion arc was 5°, the pinch strength of the injured finger was 1.0 kg, and the DASH score was 44. Therefore, the patient was treated with a third metacarpal base osteoarticular flap for the right second metacarpal head defect [2] (Figure 3). This surgery was performed under general anaesthesia, and a pneumatic tourniquet was used. A 6-cm longitudinal incision was made over the second dorsal metacarpal artery (DMA), which was selected as the vascular pedicle. We identified and released the second DMA and accompanying veins, and then incised the periosteum of the third metacarpal transversely at the level of the epiphysis, which was distal to the nutrient arteries. The metacarpal was cut carefully to avoid injury to the nutrient arteries, and the osteoarticular flap was elevated (articular flap size: 12 × 10 mm, pedicle length: 40 mm). This pedicled bone flap was then transferred to the second metacarpal head defect. A pivot point was chosen at a point along the portion of the DMA proximal to the communicating artery of the palmar metacarpal artery. The vascularised bone fragment was fixed with two K-wires (1.0 mm) (Figure 4(A)), taking care to restore the continuity and contour of the articular surface as much as possible. The tourniquet was then released to check for bone flap bleeding. No other procedure was performed at the donor site after harvesting the bone flap.

**Figure 5.**
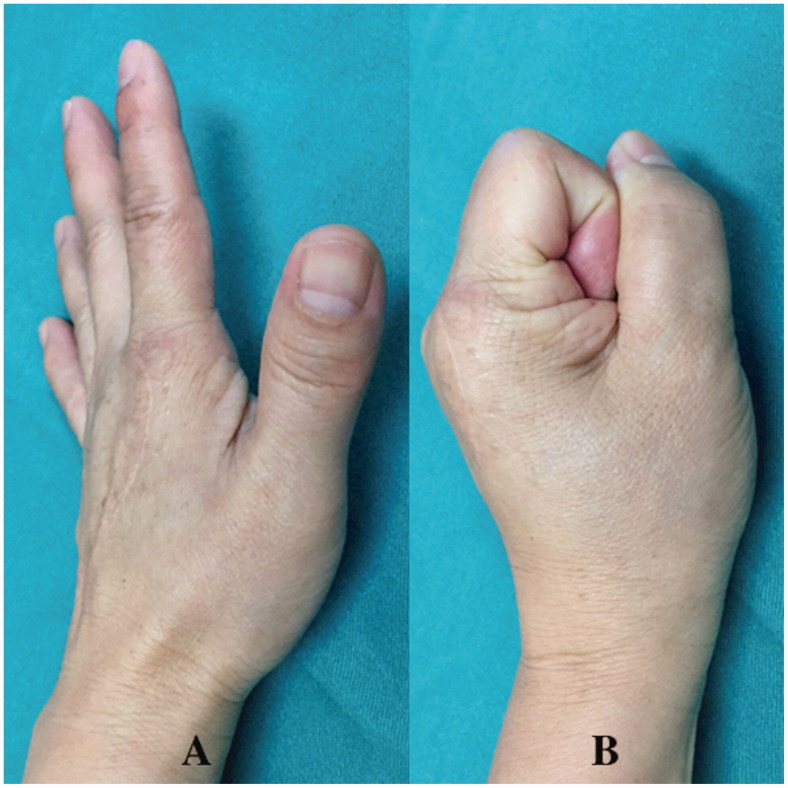
Case 1. (A and B) A good functional outcome was obtained.

A short arm splint was applied for 3 weeks postoperatively (POW 3). The K-wires fixing the vascularised bone were removed after POW 4, and active-assisted motion of fingers was then allowed. Hand therapy with active assisted motion was undergone for 6 months. Plain radiographs showed bone union after POW 8 (Figure 4(B)) and the patient returned to her work (nurse) and normal daily activity at POW 18 (Figure 5). At final follow up of 12 months postoperatively, the motion arc was 50°, the pinch strength of the injured finger was 6.5 kg, and the DASH score was 8. There was no donor site morbidity at final follow up.

### Case 2

**Figure 6.**
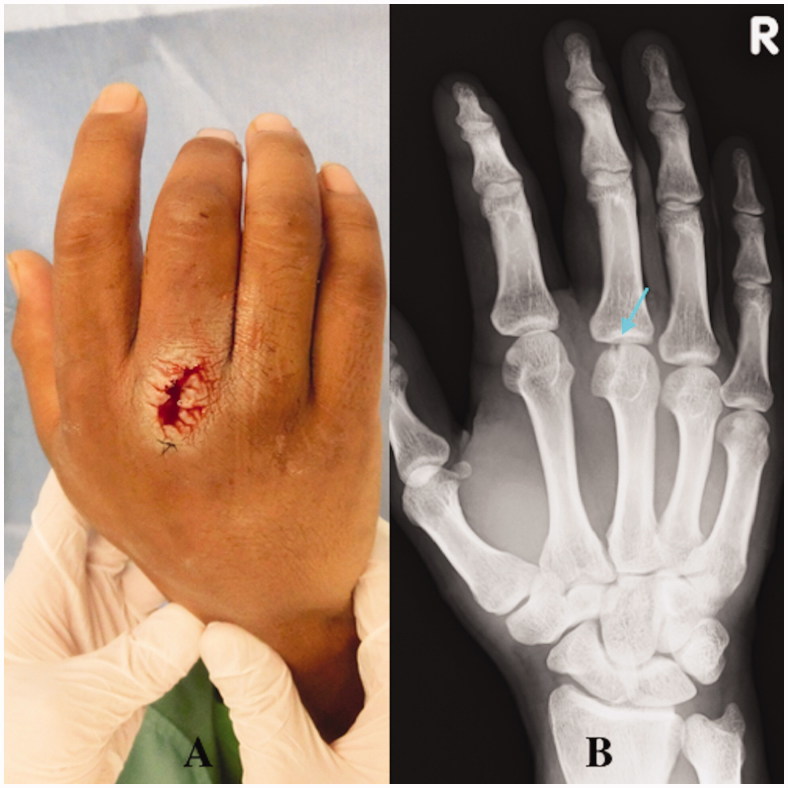
Case 2. (A) Noticeable swelling, erythema, and pus-like discharge on the dorsum of the third MCP joint. (B) The arrow shows a visible tooth sign in an oblique radiographic view.

A 32-year-old right hand-dominant man was admitted to the Department of Dermatology in our hospital with swelling and pain of the right hand. He reported an injury with a calk at work 10 days ago. A 5-mm long wound was present over the third MCP joint with redness and swelling. A dermatologist examined the patient. A simple wound dressing was applied and the patient was discharged with a prescription for regular oral antibiotics. One week later, the patient visited our hospital with noticeable swelling, erythema, tenderness, and pus-like discharge on the dorsum of his third MCP joint (Figure 6(A)). Radiographs showed a visible tooth mark at the third metacarpal head (Figure 6(B)). Body temperature was 38.5 °C. Laboratory tests revealed an increased number of white blood cells (12.00 × 10^9^/L) and elevated C-reactive protein (25.00 mg/L). We suspected osteomyelitis of the right third metacarpal head due to a clenched-fist injury and quickly performed wound exploration in the operation room. A partial lesion of the extensor and the second metacarpal head due to bite and infection was found (Figure 6(B)).

**Figure 7.**
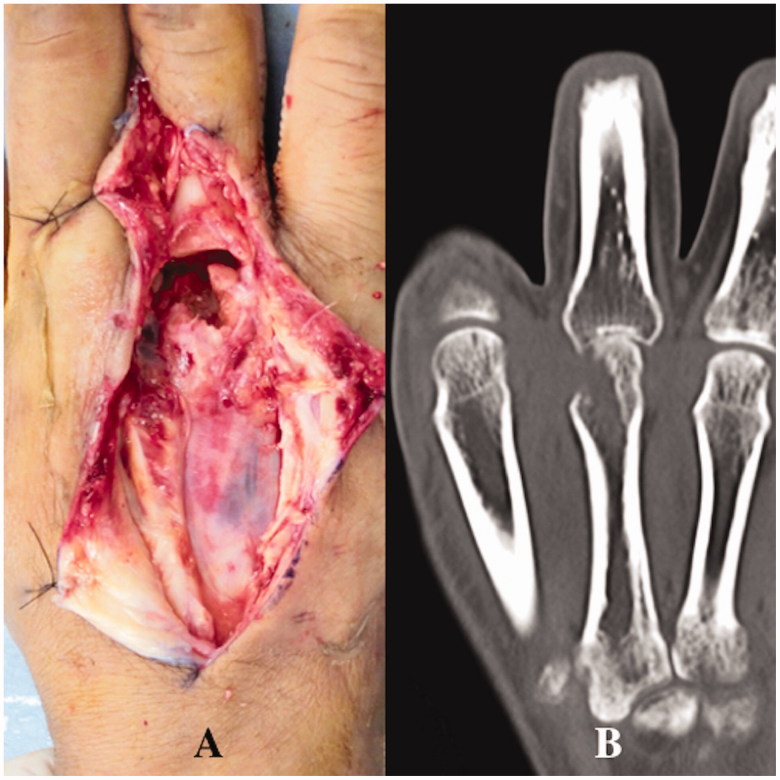
Case 2. (A) Intraoperative view showing the partial lesion of the extensor and the second metacarpal head due to bite and infection. (B) CT showing the third metacarpal head defect and the narrowed joint space.

**Figure 8.**
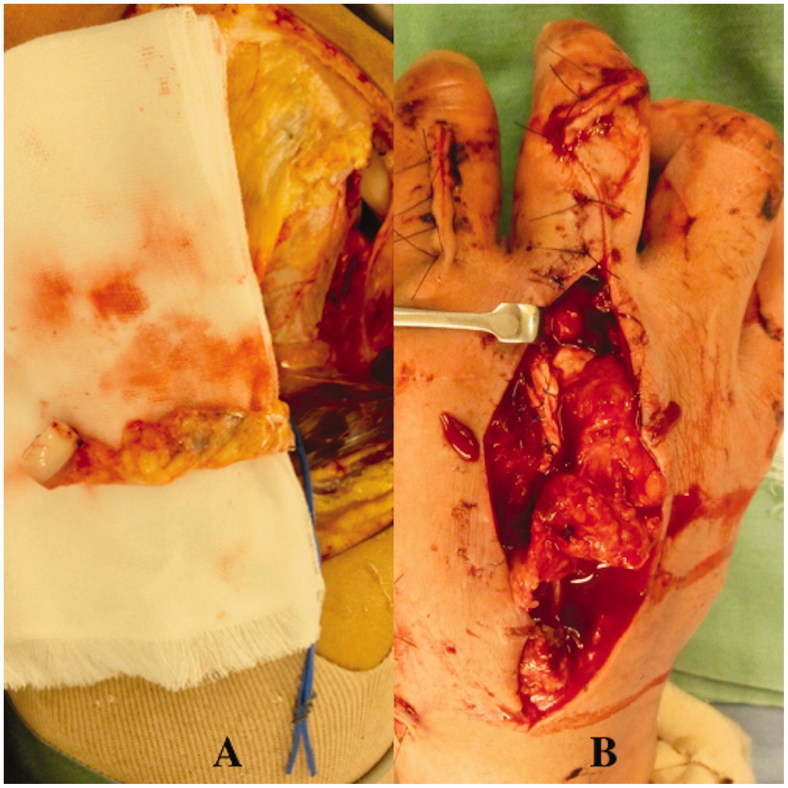
Case 2. (A) An osteochondral vascularised MFT flap was harvested. (B) The flap was transferred to the third metacarpal head defect.

**Figure 9.**
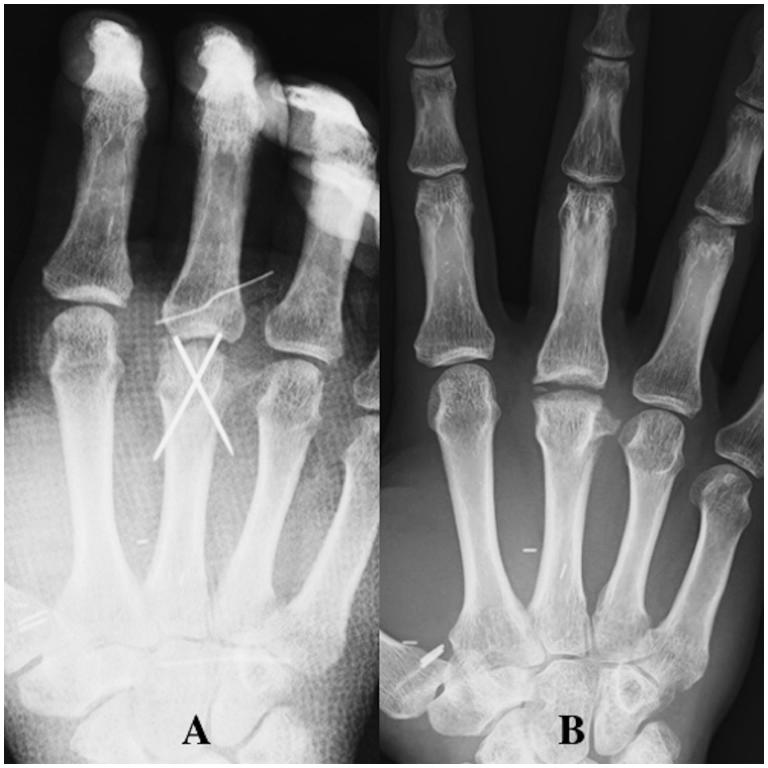
Case 2. (A) The osteochondral bone segment fixed by K-wires. (B) Radiography showed a thick layer of cartilage at the MCP joint without joint space narrowing at final follow up.

An intraoperative Gram-stain wound culture did not reveal any bacteria due to treatment with the initial oral antibiotics. Surgical debridement was performed three times with intravenous systematic antibiotic therapy to resolve osteomyelitis completely. A large defect of the third extensor and third metacarpal head occurred due to the repeated debridements (articular defect size: 18 × 15 mm) (Figure 7(A)). The patient could not move his third finger due to pain. The motion arc was 0°, the pinch strength of the injured finger was 1.0 kg, and the DASH score was 52. Therefore, the patient was treated with an osteochondral vascularised medial femoral trochlea (MFT) flap for the third metacarpal head defect (Figure 7(B)). The flap was harvested using the method described by Bürger et al. [3,4]. The width, length, and depth of the osteocartilaginous segment were 18, 15, and 12 mm respectively. This segment was harvested on the transverse branch and common descending geniculate artery (DGA) (Figure 8(A)). The length of vascular pedicle available was 6.0 cm. No other procedure was performed at the donor site after harvesting the bone flap. DGA vessels were end-to-end anastomosed to the radial artery and accompanying vein at the snuff box (Figure 8(B)). Fixation was achieved with two K-wires (1.2 mm) through a dorsal approach at the MCP joint (Figure 9(A)). The cartilage-bearing segment of the MFT provided a good counter match with the MCP joint. Third extensor reconstruction was performed with a palmaris longus tendon graft.

**Figure 10.**
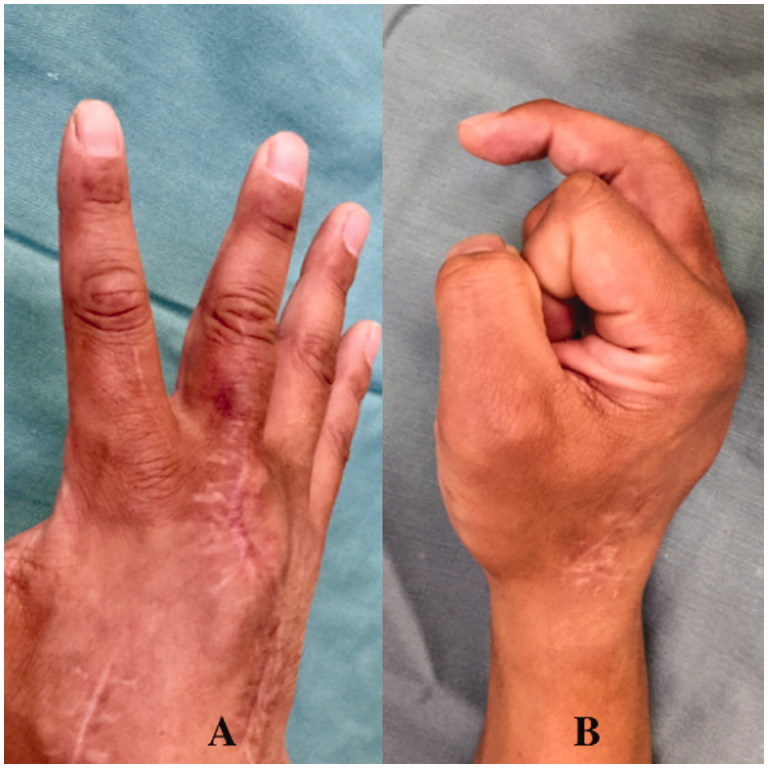
Case 2. (A and B) The motion arc was 30° at final follow up.

After surgery, the patient was immobilised in a finger splint for 4 weeks. The K-wires fixing the vascularised bone were removed after POW 8. Osseous union was detected on radiography at POW 10. Hand therapy with active assisted motion was started at 4 weeks postoperatively and undergone for 6 months. The patient returned to his work (civil engineer) and normal daily activity at 6 months postoperatively (Figure 10). At final follow up of 12 months postoperatively, the motion arc was 30° (extension lag: 4°), the pinch strength of the injured finger was 8.0 kg, and the DASH score was 13. X-ray showed a thick layer of cartilage at the MCP joint without joint space narrowing at final follow up (Figure 9(B)). There was no morbidity such as pain at the knee donor site.

## Discussion

Management of massive chondral defects remains as a significant challenge in orthopaedic surgery because joint reconstruction at the MCP level is difficult [5–7]. A free osteochondral autograft is commonly used as a conventional option for metacarpal joint reconstruction. Graft donor sources include the dorsal portion of the hamate, radial styloid, or rib cartilage, distal femur or foot [8–13]. However, joint space narrowing or cartilage degeneration, or resorption can occur in the long-term [14–17]. Higgins et al. found that osteochondral vascularised flaps in an intrasynovial environment have superior cartilage quality and survival compared with nonvascularised grafts [18], Ishii et al. showed that pedicled pisiform osteochondral transfer could restore a large cartilage defect after intra-articular distal radius fracture [6], and del Piñal et al. reported good results with a vascularised osteochondral graft from the metatarsal base in reconstruction of the articular surface of the radius [19]. Many other studies have described long-term results of vascularised osteochondral grafts with preservation of joint space and maintenance of joint architecture [7,10]. Prosthetic arthroplasty or MCP joint arthrodesis are alternative procedures, but may not necessarily produce good outcomes [20]: the former may lead to loosening of the implant and the latter results in loss of joint motion and risks of excessive bone shortening and non-union [21].

In our two cases, we performed reconstruction with vascularised osteoarticular flaps for the following reasons. First, vascularised tissue transfer is an effective treatment for infection because of the improvement of local blood flow and resulting antibiotic delivery [22]. Second, the articular cartilage can be reconstructed simultaneously [2–4]. Third, better long-term outcomes are expected with vascularised osteoarticular flaps compared to nonvascularised cartilage grafts [18]. In case 1, we used a third metacarpal base osteoarticular flap for the second metacarpal head defect. In this surgical procedure, it was easy to harvest a periosteal flap and the method is reliable due to the relatively large and anatomically constant pedicle that is long enough to reach the metacarpal head defect. The adjacent location of the donor site may also enable relatively less invasive surgery, and the advantages of this flap also include a low rate of donor-site morbidity. However, it was difficult to gain a sufficient amount of bone flap to achieve coverage for a large metacarpal head defect, and the contour match to the articular surface was very difficult with this flap. As a result, there was a 2.5-mm articular stepoff postoperatively, but good functional outcome was achieved at final follow up. This may have been due to part of the normal articular cartilage that was not damaged. In case 2, a vascularised osteocartilaginous MFT flap was used for a large third metacarpal head defect because it was difficult to reconstruct this defect with a third metacarpal base osteoarticular flap due to the defect size. In contrast, the MFT flap was easy to adjust to the defect size, and contour matching was also easy due to the original shape of the MFT flap. The disadvantages of this flap are surgical difficulty and a long operation time compared with a third metacarpal base osteoarticular flap. Although our patient reported no pain at the knee donor site, donor site morbidity may also occur using this flap [3,4].

Excellent results have been reported with use of an osteochondral vascularised MFT flap for treatment of recalcitrant proximal pole scaphoid nonunions and collapsed lunates in the setting of advanced Kienböck disease [3,4,18]. However, to the best of our knowledge, there has been no report in the English literature of use of this flap to treat a metacarpal head defect, or comparison of an osteochondral vascularised MFT flap with a third metacarpal base osteoarticular flap. Based on our two cases, the indication for treatment of a metacarpal head defect may vary according to the size of the defect and the patient’s age, occupation, and daily activities.
